# Supplementary material for: Evaluation of protein intake and protein quality in New Zealand vegans
Source: PLoS One. 2025 Apr 16;20(4):e0314889. doi: 10.1371/journal.pone.0314889 (PMC12002464; doi:10.1371/journal.pone.0314889)
Supplement: S1 File — S1 Table: Energy intake as distinguished by sex and age.1 PAL, Physical activity level. Energy requirement values are obtained from Nutrient Reference Values of Australia and New Zealand which provided low to moderate activity range (28) and based on 76 kg male and 61 kg female. Percentage adequacy is represented as individuals who are at or above the low range of energy requirements. For males, this would be 10.8 MJ (19 - 30 years), 11 MJ (31 - 50 years), 9.5 MJ (51 – 70 years), 7.4 MJ (above 70 years), and for females, this would be 8.1 MJ (19 - 30 years), 7.9 MJ (31 - 50 years), 7.6 MJ (51 – 70 years) and 7.1 MJ (above 70 years). Dash lines represent not applicable calculations as there are no males above 70 years of age in the study. S2 Table: Protein intake and body composition. n represents number of individuals. Values represented as mean (SD) unless stated. Adequacy for protein is derived by comparing individual protein intake in g/kg BW/d with the EAR provided by the Nutrient Reference Values of Australia and New Zealand (28) which is 0.60 g/kg/d for females and 0.68 g/kg/d for males, between 19 and 70 years of age, and 0.75 g/kg/d for older females. S3 Table: Energy and AMDR of individuals who were below daily requirements for lysine and leucine. n represents number of individuals. Energy requirement values are obtained from Nutrient Reference Values of Australia and New Zealand (28) and based on 76 kg male and 61 kg female. Percentage adequacy is represented as individuals who meet or are above the low range of energy requirements. For males, this would be 10.8 MJ (19 - 30 years), 11 MJ (31 - 50 years) and 9.5 MJ (51 – 70 years). For females, this would 8.1 MJ (19 - 30 years), 7.9 MJ (31 - 50 years) and 7.6 MJ (51 – 70 years). Dash lines represent not applicable calculations as there are no males above 70 years of age, and no females above 70 years of age that are inadequate for TID-adjusted lysine and leucine in the study. AMDR, Acceptable Macronutrient [file pone.0314889.s001.docx]

Supporting information

**S1 Table** Energy intake as distinguished by sex and age.

| **Sex** | **Energy requirement (MJ)** | | **Mean energy intake (MJ)** | **Percentage adequate (%)** |
| --- | --- | --- | --- | --- |
|  | Age (years) | Range from PAL^1^ of low to moderate activity (MJ) |  |  |
| Male | 19 – 30 (n = 9) | 10.8 – 13.8 | 11.2(1.9) | 55.6 |
|  | 31 – 50 (n = 29) | 11 – 16.1 | 10.7(2.2) | 37.9 |
|  | 51 – 70 (n = 14) | 9.5 – 12.1 | 9.4(3.5) | 28.6 |
|  | > 70 (n = 0) | 7.4 – 13.6 | - | - |
| Female | 19 – 30 (n = 43) | 8.1 – 10.5 | 7.5(1.6) | 27.9 |
|  | 31 – 50 (n = 65) | 7.9 – 10.1 | 7.7(2.0) | 40.0 |
|  | 51 – 70 (n = 31) | 7.6 – 9.6 | 7.0(1.6) | 35.5 |
|  | > 70 (n = 2) | 7.1 – 9.1 | 10.1(3.2) | 100 |

^1^ PAL, Physical activity level. Energy requirement values are obtained from Nutrient Reference Values of Australia and New Zealand which provided low to moderate activity range (28) and based on 76kg male and 61 kg female. Percentage adequacy is represented as individuals who are at or above the low range of energy requirements. For males, this would be 10.8 MJ (19 - 30 years), 11 MJ (31 - 50 years), 9.5 MJ (51 – 70 years), 7.4 MJ (above 70 years), and for females, this would be 8.1 MJ (19 - 30 years), 7.9 MJ (31 - 50 years), 7.6 MJ (51 – 70 years) and 7.1 MJ (above 70 years). Dash lines represent not applicable calculations as there are no males above 70 years of age in the study.

**S2 Table** Protein intake and body composition

| **Sex** | ***n*** | **Mean Energy intake**  **in MJ** | **Mean AMDR for protein in percentage** | **Mean weight in kg** | **Mean BMI**  **in kg/m^2^** | **Mean body fat in percentage** |
| --- | --- | --- | --- | --- | --- | --- |
| **Adequate for protein** | | | | | | |
| Female | 103 | 7.9 (1.8) | 12.8 (3.9) | 63.7 (8.6) | 22.9 (2.7) | 31.8 (5.7) |
| Male | 41 | 11.0 (2.4) | 12.8 (3.6) | 77.0 (11.1) | 24.1 (2.8) | 22.0 (4.8) |
| **Inadequate for protein** | | | | | | |
| Female | 38 | 6.3 (1.4) | 9.9 (1.9) | 72.1 (12.6) | 26.0 (3.4) | 37.5 (4.8) |
| Male | 11 | 8.1 (1.7) | 10.3 (1.8) | 88.4 (13.5) | 26.5 (3.2) | 26.1 (4.4) |

*n* represents number of individuals. Values represented as mean (SD) unless stated. Adequacy for protein is derived by comparing individual protein intake in g/kg BW/d with the EAR provided by the Nutrient Reference Values of Australia and New Zealand (28) which is 0.60 g/kg/d for females and 0.68 g/kg/d for males, between 19 and 70 years of age, and 0.75 g/kg/d for older females.

**S3 Table** Energy and AMDR of individuals who were below daily requirements for lysine and leucine

|  |  | **Male** | | | **Female** | | | |
| --- | --- | --- | --- | --- | --- | --- | --- | --- |
| Age (years) | *n* | Mean Energy intake in MJ | Percentage adequate for energy in % | Mean AMDR (Percentage adequacy in %) | *n* | Mean Energy intake in MJ | Percentage adequate for energy in % | Mean AMDR (Percentage adequacy in %) |
|  | **Inadequate for lysine** | | | | | | | |
| 19 - 30 | 3 | 9.6 | 0 | 10.2 (33.3) | 22 | 6.7 | 9.1 | 10.5 (59.1) |
| 31 – 50 | 15 | 9.4 | 20 | 10.6 (73.3) | 40 | 7.5 | 37.5 | 10.6 (57.5) |
| 51 – 70 | 7 | 7.8 | 14.3 | 10.4 (71.4) | 22 | 6.6 | 27.3 | 10.7 (68.2) |
| > 70 | - | - | - | - | - | - | - | - |
|  | **Inadequate for leucine** | | | | | | | |
| 19 - 30 | 1 | 9.8 | 0 | 9.8 (0) | 18 | 6.5 | 5.6 | 10.2 (50.0) |
| 31 – 50 | 10 | 9.2 | 10 | 11.1 (70.0) | 30 | 7.2 | 26.7 | 10.3 (50.0) |
| 51 – 70 | 6 | 7.3 | 0 | 10.5 (83.3) | 20 | 6.4 | 20.0 | 10.8 (70.0) |
| > 70 | - | - | - | - | - | - | - | - |

*n* represents number of individuals. Energy requirement values are obtained from Nutrient Reference Values of Australia and New Zealand (28) and based on 76kg male and 61 kg female. Percentage adequacy is represented as individuals who meet or are above the low range of energy requirements. For males, this would be 10.8 MJ (19 - 30 years), 11 MJ (31 - 50 years) and 9.5 MJ (51 – 70 years). For females, this would 8.1 MJ (19 - 30 years), 7.9 MJ (31 - 50 years) and 7.6 MJ (51 – 70 years). Dash lines represent not applicable calculations as there are no males above 70 years of age, and no females above 70 years of age that are inadequate for TID-adjusted lysine and leucine in the study. AMDR, Acceptable Macronutrient Distribution Range is for protein, which is between 10 to 35% of caloric intake. (26) Percentage adequacy is represented as individuals who meet or are above the lowest range of AMDR for protein, 10%
