# Supplementary material for: Evaluation of protein intake and protein quality in New Zealand vegans
Source: PLoS One. 2025 Apr 16;20(4):e0314889. doi: 10.1371/journal.pone.0314889 (PMC12002464; doi:10.1371/journal.pone.0314889)
Supplement: Attachment — Submitted filename: Response to Reviewers.docx [file pone.0314889.s003.docx]

13^th^ February 2025

Manuscript ID: PONE-D-24-52340

Manuscript Title: Evaluation of protein intake and protein quality in New Zealand vegans

Dear Editor

We wish to submit a substantially revised version of our original research for the consideration by PLOS ONE. The enclosed work is not under consideration for publication elsewhere.

We thank all reviewers for the thoroughness in reviewing the manuscript and providing constructive feedback which could only serve to improve the quality of the paper. All comments have been taken into consideration and necessary changes have been implemented. Please see the response to each point in the table below.

| **Reviewer comments** | **Author response** | **Page and line number of revisions** |
| --- | --- | --- |
| 1. It starts by saying that only 0.74% of the population in the NZ Health Survey identifies as vegan. It goes on to say that “some studies” report a growing trend among young adults and a greater popularity of plant-based diets among women. It’s unclear: is veganism really growing, or is it just plant-based diets in general? Is the New Zealand data representative of the global trend? | The text has been revised to segregate trends of vegan diet adoption globally and in NZ. We highlight that although a vegan diet is not normative in NZ, potential nutrient inadequacies as a source of concern is worth investigating and addressed this to be a more prominent problem in unbalanced diets. | Page 2, lines 47-50 |
| 2. “Concerns on nutrient inadequacies accorded by a vegan diet have been highlighted” – awkward wording, better: Concerns about nutrient inadequacies in a vegan diet have been highlighted. | The sentence has been reconstructed for improved coherence. | Page 2, lines 49-50 |
| 3. it mentions “long-chain fatty acids such as docosahexaenoic acid” (DHA). However, DHA is not a typical fatty acid in plant foods, and its deficiency is more likely due to a lack of algal sources or supplementation. | Fatty acids, if included would warrant a detailed discussion and thus, this point is removed from the revised manuscript as fatty acids are not the nutrient of focus in this research. |  |
| 4a. The claim that “regular high-quality protein intake throughout the day is essential to ensure IAA quantities are at threshold level” suggests that only high-quality protein can provide adequate amounts of essential amino acids. In fact, if the diet is well-balanced, various plant protein sources can complement each other’s amino acid profiles.  4b. There is also no emphasis on the fact that plant proteins can complement each other, which means that a well-composed vegan diet can provide all the necessary amino acids. | Since plant proteins are considered to have lower protein quality as compared to animal-sourced proteins (as mentioned in the paragraph), the subsequent use of high-quality protein would be inappropriate. The text has now addressed the approach of simultaneous provisions of complementary plant proteins, at least within a vegan diet context, to improve IAA consumption. | Page 4, lines 73-77  Page 5, line 84  Further emphasis present in the original text (Page 4, lines 95-100; Page 26, lines 517-518  Additional emphasis provided in revised text Page 3, lines 57-59; Page 4, lines 73-77, 84; Page 28, lines 562-565 |
| 5. It first states that “few studies evaluated amino acid (AA) intake among vegans,” and later that “diet consisting solely of plant proteins typically provides lower amounts of digestible amino acids.” Since few studies have evaluated amino acid intake among vegans, how can one claim that their intake is inadequate? | We would like to clarify that there is a distinction between the research on intake patterns of vegan diets and the known digestibility of plant proteins. While relatively few studies have analysed amino acid composition in vegan diets and even fewer have accounted for digestibility of plant foods within the context of vegan diets, this does not contradict the established fact of plant proteins having lower digestibility. One evidence can be provided by lower DIAAS scores from plant proteins. The statement about plant protein digestibility is not an assumption of vegan diet patterns, but stating the intrinsic properties of plant proteins. The evaluation of AA intake among vegans and whether they meet daily requirements will depend on food choices and combinations. Hence, the two statements are separate discussions. |  |
| 6a. “Within a meal context, the presence of at least one limiting IAA halts protein synthesis” is an oversimplification. Metabolic processes do not immediately stop because of one missing amino acid.  6b. The statement: "Presence of at least one limiting IAA halts protein synthesis and deposition within the body" suggests that protein synthesis stops completely when one amino acid is missing. In fact, the body may be partially adjusting metabolism by recycling amino acids or limiting muscle catabolism. | We acknowledge the oversimplification of metabolic processes in this statement and have made improvements in the revised manuscript, following checks in more recent literature. The included text now provides a general discussion on various factors influencing muscle protein synthesis.  A recent study discussed the possibility of compensation of missing amino acids by plasma reserves, but this mechanism has not been proven in humans, and is limited in pigs.  Doi: <https://doi.org/10.1016/j.cdnut.2024.104439> | Page 4, lines 79-87  Page 4, lines 67-68 |
| 7. "The main aim of this study was to determine protein adequacy among a cohort of vegans in NZ" – but in what aspect? Only quantitative (grams of protein) or also qualitative (amino acid profile, digestibility)? | Included this suggestion in the revised manuscript | Page 5, lines 107-110; |
| 8. In the first sentence, veganism is defined as a diet that excludes animal products, but later the term "plant-based diet" (PB diet) is used, which can also include flexitarian or vegetarian diets. It would be necessary to clarify whether PB diet in this text means a strictly vegan diet or a more general plant-based approach. | We agree that a vegan diet should be clearly distinguished with other plant-based diets and have added this revision in the manuscript with the appropriate definitions. Following these definitions, the text only uses the term “vegan diet”. | Page 2, lines 38-41 |
| 9. The text indicates that plant sources of protein have lower protein quality due to the presence of limiting amino acids (IAAs) and anti-nutritional factors. However, there is no explanation as to whether proper planning of a vegan diet actually leads to deficiencies or only increases the risk of their occurrence. | This has been corrected in the revised manuscript. | Page 4, lines 73-74 |
| 10. Food amino acid data were obtained from the USDA database, although the study was conducted in New Zealand. It was not stated whether local products differ significantly in composition, nor whether other databases are available that are adapted to the actual foods consumed in New Zealand. | The best available database used to obtain amino acid data was from the USDA as the NZ food database does not contain amino acid values. We have checked for anomalies in the protein content between the USDA and NZ FOODfiles. |  |
| 11. Normalizing amino acid values from the USDA to the FOODfiles database may lead to errors in estimates if the differences in composition are significant. |  |  |
| 12. The decision to exclude all products providing <0.5 g protein may lead to underestimation of protein and amino acid intake, especially if these foods are consumed frequently. | We clarify that the 0.5g was based on crude protein and not digestibility-adjusted protein to err on the side of caution in removing excessive food items. For foods or ingredients that were part of a recipe, they were not excluded even if they had protein content < 0.5g, because these ingredients can add up to substantial quantities of protein. Only complete recipes with < 0.5g were removed. The decision to remove foods < 0.5g was to condense the number of food items for analysis. A review of the removed food items showed that these were mainly water, beverages such as tea, soda, condiments (salt and spices), and fruit which were also not found to be high protein contributors in the diet, as shown in Figure 7. The exclusion of foods < 0.5g still met close to 100% of total protein provision by the vegan diet, as explained in lines 171-172. | Added explanation: Page 8, lines 170 |
| 13. A protein quality assessment based on the ratio of IAA (mg) to total protein intake is described. However, no mention was made of more standard methods such as PDCAAS (Protein Digestibility Corrected Amino Acid Score) or DIAAS (Digestible Indispensable Amino Acid Score), which are commonly used to assess protein quality. | Indeed, PDCAAS and DIAAS can be used to measure utilisable proteins in single food products. The application of PDCAAS has several known limitations, especially that of truncation and the use of faecal rather than true ileal digestibility. While DIAAS is noted to be a more superior measurement for protein quality, it is limited to single foods rather than a mixed diet setting of this cohort. At the point of this research, the TID values for many plant-based foods (as highlighted in our limitations; lines 600-601) are absent and would not translate to accurate DIAAS measurements. Additionally, DIAAS identifies the score of the first limiting IAA, and in the context of a whole diet, may not provide sufficient information about other limiting IAAs if present. To meet the objective of the study, we aimed to compare the current intake of protein and IAA with the WHO/FAO daily requirements to assess how much the vegan diet can provide for protein quantity and quality as compared to the requirements. | Page 10, lines 187-198 |
| 14. A definition of normal BMI for the New Zealand population is provided, but it is worth noting that standards may differ for different ethnic groups (e.g. people of Asian descent may have different BMI thresholds for overweight and obesity). | The cohort population was mostly NZ of European descent although there were individuals of different ethnicities. In the revised manuscript, this has been addressed in limitations. | Page 31, lines 641-643 |
| 15. For body fat percentage, thresholds for overweight and obesity are provided, but no reference is made to standards for normal body fat. | Few published studies have examined or provided normal body fat percentages, with BMI typically being used as indications for normal and excessive body fat, even in the NZ Eating and Activity guidelines by the Ministry of Health. We have not examined detailed relationships between anthropometrics and protein adequacy in this research as this is not one of our main objectives. However, further checks in the current literature provided a normal body fat percentage that we can use as an indication – we have added this in the revised manuscript. | Page 10, lines 202-204 |
| 16. There is no mention of possible errors in measuring dietary intake (e.g. underestimation in food diaries), or of the limitations of using a single DXA body composition measurement. If the mean protein intake meets the EAR for both sexes, but only 56.2% meet the essential amino acid requirements, this suggests a serious protein quality problem. However, later on, it is stated that “meeting protein quality in a vegan diet can be likely achieved when total protein requirement is met” (lines 505–507), which contradicts the previous conclusion. | We have mentioned potential errors with the use of the four-day food diary in terms of underreporting and drawn links with the consequence of low energy intake within the cohort (page 30, lines 613-615).  For limitation with DXA, we have added this to the revised manuscript.  In the second statement / conclusion about “meeting protein quality in a vegan diet can be likely achieved…” refers to Figure 6 where the numbers reflect individuals that could meet at least one of the IAAs, while the previous statement was a conclusion based on meeting all IAAs. We acknowledge this contradictory statement and the confusion it caused and have rectified this in the manuscript. | Page 31, lines 640-641  Page 28, lines 544-549 |
| 17. If the group had a higher body mass and at the same time consumed less energy, then either there is a serious error in the measurements (e.g. underreporting of energy intake) or these individuals must have had a lower metabolic rate, which is not explained. | Additional explanations are made in the revised manuscript. | Page 25 and 26, lines 475-480 |
| 18. It is suggested here that people with a normal BMI meet the amino acid requirements more easily, but a few lines later (lines 435–438) it is stated that people with a higher BMI may have a lower protein requirement. There is no clear explanation of exactly how body composition affects protein requirements. | We indicated that a normal BMI is an indication of healthy or lower body weight. We have improved the text for clarity to explain the statement. | Page 25, lines 478-481 |
| 19. lines 505–507 If so, why was it previously found that 43.8% of individuals did not meet the standards for IAAs despite having adequate protein intake? This suggests that protein quantity alone does not guarantee protein quality, so this paradox needs to be better explained. | Lines 505-507 in the original manuscript stated: *“Overall, we have shown that, while the majority of vegans in the cohort were meeting total protein intake compared to the EAR, about a quarter had insufficient intake (****Table 2****)”* . The insufficiency here refers to overall protein intake and not the IAA. Further explanation provided in revised manuscript. | Page 29, lines 556-565 |
| 20. Although it is a small percentage worldwide, it is important to generate knowledge that allows to know that their diet has an optimal quality. |  | Added to conclusion, page 34, lines 674-676 |
| 21. The main observation made by this reviewer when reading this manuscript was the processing and collection of data. The dietary record may lack reliability, since the proportions may be underestimated, or not report all the foods consumed due to forgetfulness or fatigue. On the other hand, not having indicators specific to the sample under study and reusing those used in other populations can be a disadvantage.  Although the aforementioned may be a disadvantage of the data presented in this study, the authors take it up in the limitations section and provide valid arguments for the conclusions. | Rechecked and improved limitations section |  |
| 22. Revise the wording of “Data processing” as it is confusing. | Wording has been modified | Page 7, Line 141 |

Additional requirements and comments:

- We noted small discrepancies in our raw data and conducted recalculations that are reflected in the manuscript, such as in the tables. There are no major differences in values, and the changes do not alter the final interpretation and conclusions of the paper. The entire paper has been checked again and updated accordingly.
- Comment 1: In accordance to PLOS ONE’s style requirements, this has been updated
- Comment 2: Funding information has been provided in updated cover letter
- Comment 3: In accordance with the guidelines from PLOS ONE Data Policy, the personal information from the supporting information (in Minimal dataset) have been removed. The removed information are: unique identifying numbers, sex, age, ethnicity, anthropometry measurements and education
